# Floral Scent Chemistry of *Luculia yunnanensis* (Rubiaceae), a Species Endemic to China with Sweetly Fragrant Flowers

**DOI:** 10.3390/molecules22060879

**Published:** 2017-05-25

**Authors:** Yuying Li, Youming Wan, Zhenghai Sun, Taiqiang Li, Xiongfang Liu, Hong Ma, Xiuxian Liu, Rui He, Yan Ma, Zhenghong Li

**Affiliations:** 1 Research Institute of Resource Insects, Chinese Academy of Forestry, Kunming 650224, China; liyuy_ing@163.com (Y.L.); wanyouming@126.com (Y.W.); li_taiqiang@163.com (T.L.); liuxiongfang16@163.com (X.L.); lxx205@126.com (X.L.); herui61@aliyun.com (R.H.); iccxmz@aliyun.com (Y.M.); 2 School of Gardening, Southwest Forestry University, Kunming 650224, China; sunzhenghai1978@163.com

**Keywords:** *Luculia*, volatile organic compound, SPME-GC-MS, floral scent, flower development

## Abstract

*Luculia* plants are famed ornamentals with sweetly fragrant flowers. *Luculia yunnanensis* Hu is an endemic plant from Yunnan Province, China. Headspace-solid phase microextraction-gas chromatography-mass spectrometry (HS-SPME-GC-MS) was used to identify the volatile organic compounds (VOCs) of the different flower development stages of *L. yunnanensis* for the evaluation of floral volatile polymorphism. The results showed that a total of 40 compounds were identified at four different stages. The main aroma-active compounds were 3-carene, α-cubebene, α-copaene, δ-cadinene, and isoledene. Floral scent emission had the tendency to ascend first and descend in succession, reaching its peak level at the initial-flowering stage. The richest diversity of floral volatiles was detected at the full-flowering stage. Principal component analysis (PCA) indicated that the composition and its relative content of floral scent differed at the whole flower development stage. In comparison with the other two species of *Luculia* (*L. pinceana* and *L. gratissima*), the composition and its relative content of floral scent were also different among the tree species.

## 1. Introduction

The genus of *Luculia* Sweet comprises small trees or shrubs belonging to the Rubiaceae family (Trib. Cinchoneae). It has about five species in the world, mainly distributed in Southeastern Asia. There are three species distributed in China; i.e., *L. pinceana* Hooker, *L. gratissima* (Wallich) Sweet and *L. yunnanensis* Hu. Among them, *L. yunnanensis* is the unique one, endemic to Yunnan Province and naturally confined to open slopes and secondary shrubby woodland on limestone mountains at an altitude between 1200 and 3200 m [1]. *Luculia* species can be easily recognized by the compact and long-term blooming inflorescences consisting of white, pink to red, and sweetly fragrant flowers with extremely long corolla tubes [2]. *L. yunnanensis* distinguishes itself from its allied species by the surface of inflorescence axes, the hypanthium portion of the calyx, and its fruit covered by densely tomentose pubescences. Moreover, it is difficult to find wild populations of *L. yunnanensis*, as its appropriate habitats have been fragmented by human activities [1,3].

Plants synthesize a wide variety of volatile organic compounds (VOCs) that facilitate interactions with their lived environment: attracting pollinators, protecting flowers from harmful insects, and communicating with other plants. The floral scent compositions of *L. pinceana* and *L. gratissima* have been reported [4,5]. The main components of floral scent are paeonol and γ-murolene in *L. pinceana* and *L. gratissima*, respectively. Therefore, detecting the VOCs of *L. yunnanensis* could not only give clues to its conservation biology, but also provide reference to the development of *L. yunnanensis* as an ornamental with fragrant scent in the future.

Though the species of family Rubiaceae are not common aromatic plants, *Luculia* plants have a promising prospect as an essential oil source. Components and proportions of VOCs may vary at different flower developmental stages. Therefore, it is essential to investigate the VOCs polymorphism of different stages of flower development for determination of the suitable period for *L. yunnanensis* flower harvest.

Headspace solid phase micro-extraction (HS-SPME) followed by capillary gas-chromatography mass-spectrometry (GC-MS) has high reproducibility under the same test conditions, and is currently a widely used technique for flower volatile analysis [6,7]. In this paper, we investigated the floral volatiles in *L. yunnanensis* using HS-SPME coupled with GC-MS to evaluate the volatile polymorphism of different flower development stages, which provide guidance for the evaluation of flower scent quality and the generation of fragrant *L. yunnanensis* for future breeding programs and the function of floral fragrance.

## 2. Results and Discussion

### 2.1. Identification of Scent Components

**Figure 1:**
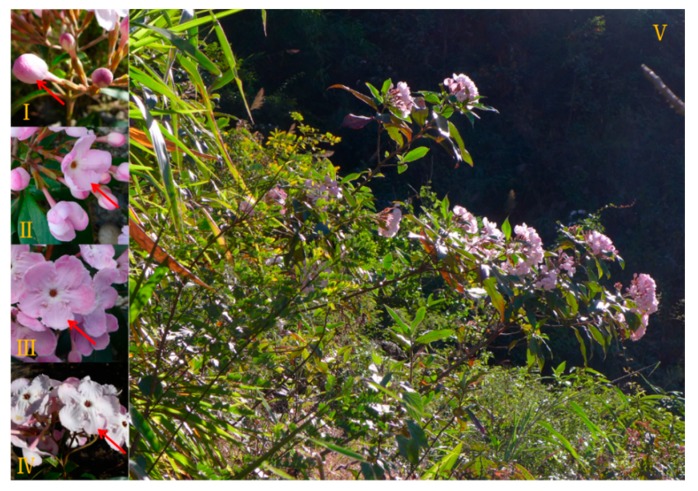
Different development stages of *L. yunnanensis* and its habitat. (I) bud stage; (II) initial-flowering stage; (III) full-flowering stage; (IV) end-flower stage; (V) habitat of *L. yunnanensis*.

The volatiles of four different stages of *L. yunnanensis* flower development (Figure 1) are given in Table 1, with the compounds listed in order of their retention time (RT) in the DB-5MS column. A total of forty VOCs were detected in *L. yunnanensis* flower development. Among these volatiles, 22 compounds were identified in four different stages. The main aroma-active compounds were identified at 3-carene, α-cubebene, α-copaene, δ-cadinene, and isoledene; these compounds might dominate the fragrance of *L. yunnanensis*. For instance, 3-carene smells like lemon or resin, α-cubebene smells like herb or wax, α-copaene smells like wood or spice, δ-cadinene smells like thyme and medicine or wood [8]. Together, the volatile compounds form the delightful fragrance of the *L. yunnanensis* flower.

**Figure 2:**
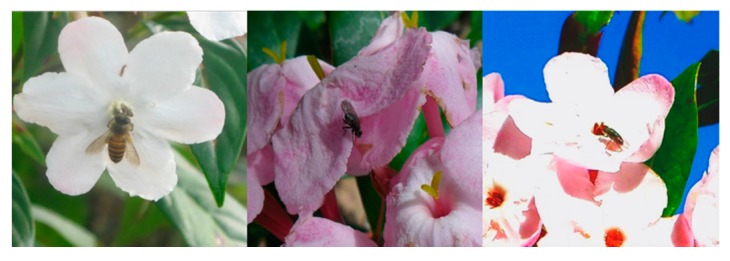
Flowers of *L. yunnanensis* visited by different insects.

As we all know, floral scent often serves as an olfactory signal for the attraction of insects. For example, bees can find the location of flowers under the guidance of linalool or 2-phenylethanol [9]; and indole for flies [10]; phenylacetaldehyde, 2-phenylethanol, oxoisophorone, linalool, and linalool oxide for butterflies [11], etc. Most visitors successfully transfer the pollen to conspecific flowers as pollinators [12]. To survive biotic stresses, many plant species have developed elaborate mechanisms to protect themselves against insects (e.g., emitting floral VOCs). (*E*,*E*)-α-Farnesene emitted from the flowers of *Prorhinotermes canalifrons* functioned as an alarm pheromone [13]. Besides (*E*,*E*)-α-farnesene, *trans*-β-ocimene (which can elicit strong antennal responses in butterfly [14]) also appeared in *L. yunnanensis*. Our preliminary results showed that the visiting insects of *L. yunnanensis* flowers in the field included *Apis florae*, *Bombus* sp., *Lucillia* sp., etc. (Figure 2).

In addition, some compounds of floral VOCs had a good effect on resisting bacteria and fungus [7,16,17]. Amongst the floral scent composition of *L. yunnanensis*, phenylethyl alcohol has antifungal activity against *Botrytis cinerea* [18]; limonene oxide has antibacterial activities against *Agrobacterium tumefaciens*, *Erwinia ananas*, *Erwinia chrysanthemi*, and *Enterobacter cloacae* [19]; paeonol has potential acaricide activity for the control of *Dermatophagoides pteronyssinus* and *Dermatophagoides farina* [16]. From this point of view, *L. yunnanensis* might be qualified as an indoor fragrant houseplant. Recent studies have shown that plants can rapidly alter their own floral volatile production in response to floral volatile cues from their neighbors in flower opening [20,21]. This specific mechanism can increase the rate of pollination and mating by increasing floral volatile emission to attract more pollinators. The relationship between the floral VOCs of *L. yunnanensis* and its environment needs further research in the future.

### 2.2. Changes of Scent Emission in Four Different Stages

**Figure 3:**
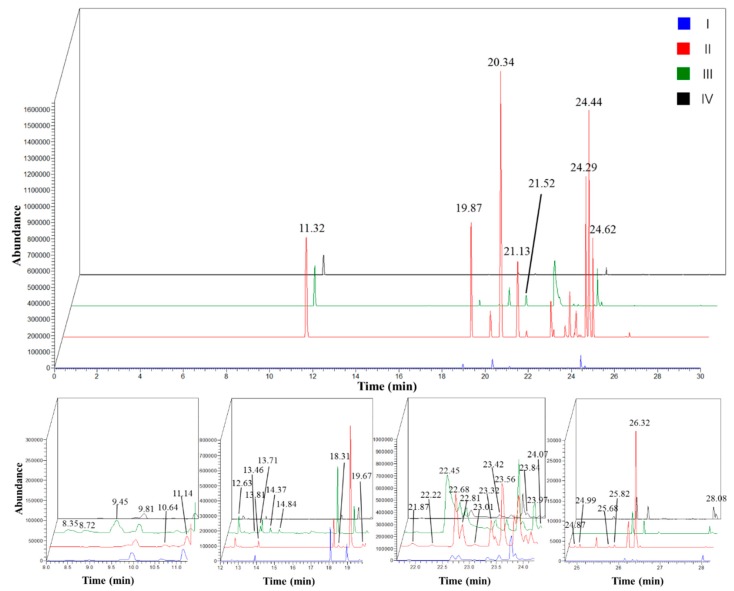
Total ionic chromatogram of scent components emitted from the flowers of *L. yunnanensis* in different stages. (I) bud stage; (II) initial-flowering stage; (III) full-flowering stage; and (IV) end-flower stage.

*L. yunnanensis* flowers were selected on the basis of their botanical characteristics to evaluate floral volatile polymorphisms according to different development stages: bud stage, initial-flowering stage, full-flowering stage, and end-flowering stage. The floral scents are orchestrated during the flower development. Table 1 and Figure 3 show the distinct changes in scent composition and concentration across flowering stages. Scent components were emitted drastically at the initial-flowering stage. The highest diversity of floral volatiles was detected at the full-flowering stage. In most plants (e.g., *citrus* flowers [22], *Vanda* Mimi Palmer [23], *Cananga odorata* [24], *Ocimum citriodorum* [25], *Penstemon digitalis* [26], and *Hosta* flowers [27]), the amount of scent emission and the diversity of floral volatiles were sharply increased at the stage of full bloom and decreased highly after full bloom. By contrast, the patterns of scent emission across *L. yunnanensis* flowering stages are different from one other.

As for the bud stage, 24 volatile compounds were identified, and the most abundant compounds were α-copaene (16.59%), δ-cadinene (14.66%), α-cubebene (13.39%), and isoledene (9.79%). For the initial-flowering stage, 31 volatile compounds were identified, and the most abundant compounds were α-copaene (17.92%), δ-cadinene (12.68%), 3-carene (10.72%), and α-cubebene (10.24%). For the full-flowering stage, 37 volatile compounds were identified, and the most abundant compounds were δ-cadinene (13.07%), paeonol (12.73%), α-copaene (9.30%), and 3-carene (9.17%). In the end-flowering stage, 26 volatile compounds were identified, and the abundant compounds were 3-carene (29.18%), humulene (9.69%), and δ-cadinene (9.16%).

The mean Bray–Curtis similarity index range was 64.29–81.40% (Table 2). The bud stage was more similar to the initial-flowering stage (*BCS* = 81.40%) than to the full-flowering stage (*BCS* = 71.88%), and was largely dissimilar to the end-flowering stage (*BCS* = 63.71%). Across all flower-life stages, the end-flowering stage was largely dissimilar to other three stages. Regarding the comparison among the studied stages, 22 volatiles among the total volatile constituents notably existed in all life-flower stages. The results showed high similarity among the four stages, although several constitutes (α-pinene, α-campholenal, phenylethyl alcohol, paeonol, etc.) were exclusively identified in different stages.

**Figure 4:**
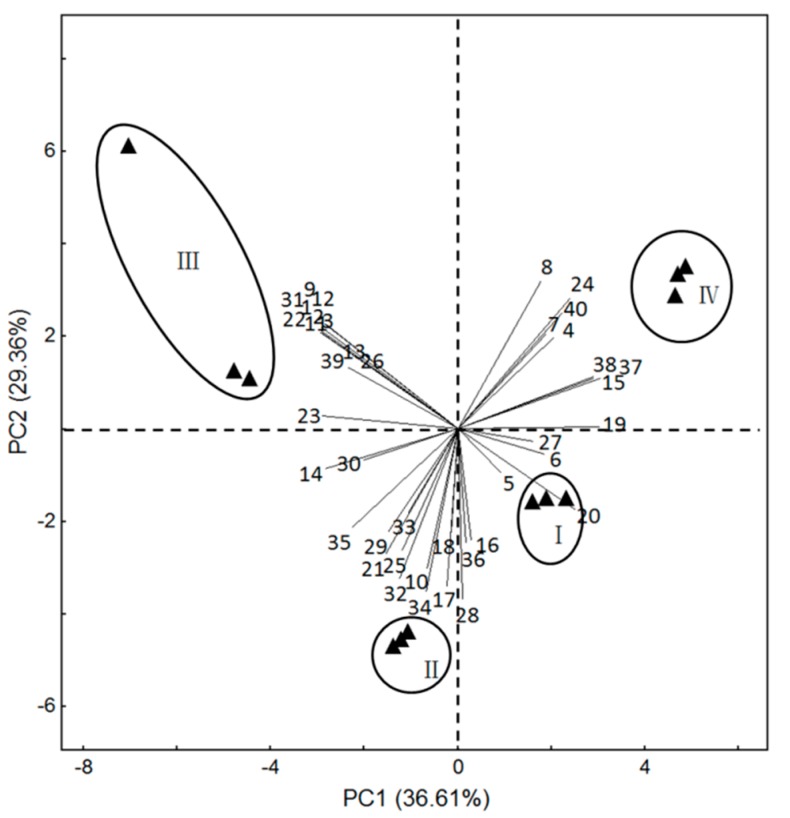
Principal component plot (PC1 vs. PC2 plots) for *L. yunnanensis* at different stages of growth, showing correlations with volatiles (numbers correspond to those in Table 1). (I) bud stage; (II) initial-flowering stage; (III) full-flowering stage; (IV) end-flower stage.

To identify which volatiles contributed the most to the differences among the four flower stages, the data on 40 volatile compounds identified in *L. yunnanensis* at whole life-flower scale were analyzed by using principal component analysis (PCA). The first two components of PCA explained 36.61% and 29.36% of the variation, explaining ~66% of combined variance (Figure 4). The volatiles that had high positive scores on PC 1 included propanoic acid, 2-methyl-, 2-ethyl-3-hydroxyhexyl ester, caryophyllene, α-acorenol, guaiol, and β-ylangene, which were highly positively related to the bud stage and the initial-flowering stage. Volatiles with high positive scores on PC 2 comprised *trans*-β-ocimene, humulene, isopropyl myristate, α-campholenal, and α-santoline alcohol, which were positively correlated with the full-flowering stage and initial-flowering stage.

### 2.3. Variation in Volatile Compounds from Three Species of Luculia

**Figure 5:**
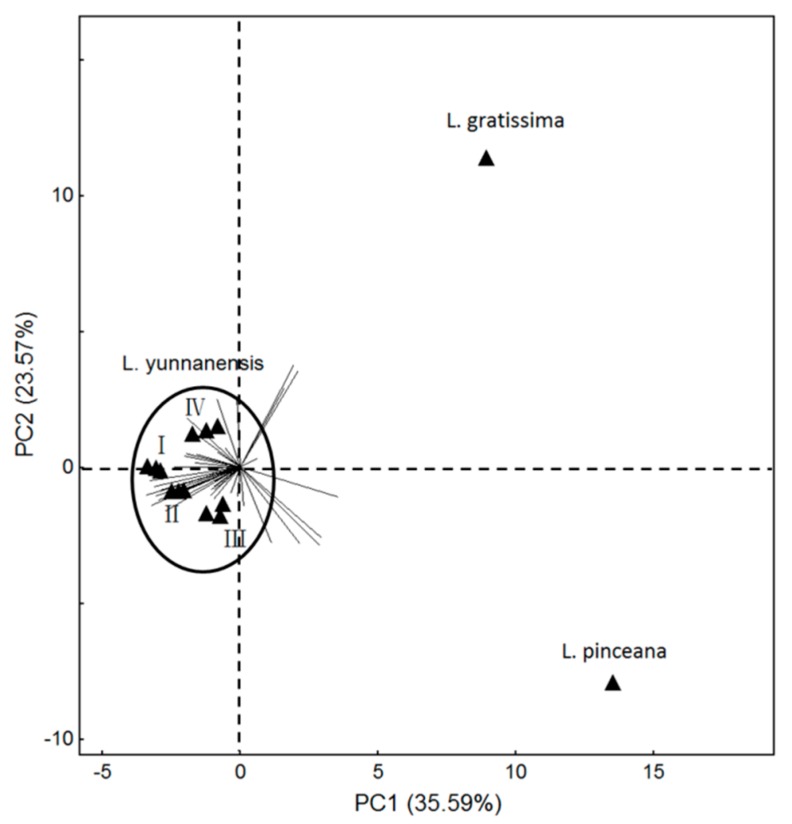
Principal component plots (PC1 vs. PC2 plots) for the volatiles of the full-flowering stage of *L. gratissima*, the full-flowering stage of *L. pinceana*, and the different flower stages of *L. yunnanensis*. (I) bud stage; (II) initial-flowering stage; (III) full-flowering stage; (IV) end-flower stage.

Sesquiterpenes were the most abundant amongst floral scent compounds, the content of which reached as high as 64% in *L. yunnanensis* and *L. gratissima* [5], in the floral scent of *L. pinceana* were benzenoids [4]. By PCA analysis, the floral scent showed differences among *Luculia* (the wide-open flower of *L. gratissima*, the full-flowering stage of *L. pinceana*, and the four stages of *L. yunnanensis*) (Figure 5). *L. gratissima* occupied 10 special compounds, such as octanoic acid, ethyl ester, β-bourbonene, *trans*-isoeugenol, etc. *L. pinceana* had 13 special compounds, such as cyclosativene, limonene, (*Z*)-verbenol, etc. *L. yunnanensis* possessed 17 special compounds, such as β-pinene, phenylethyl alcohol, limonene oxide, etc. Previous research indicated that significant differences in floral scent composition were found in closely-related species [28], which is consistent with our study.

Reports have revealed that some characterized compounds were usually rich in the early stage of flower development; for example, linalool dominated the samples from younger-stage inflorescences in *Protea* species [29], and 1,8-cineole and (*Z*)-3-hexenol were only identified from essential oil in the bud stage of *Rosa canina* [30]. These characterized compounds could make the young organs healthier [31]. However, these compounds did not appear in *L. yunnanensis* or *L. pinceana*, and this might be because the content of these volatile compounds in a single flower was too low.

## 3. Materials and Methods

### 3.1. Plant Materials

Nine fresh early flowering inflorescences of three *L. yunnanensis* plants (separate distance among plants more than 100 m, three inflorescences per plant) were collected from Nujiang Lisu Autonomous Prefecture, Yunnan Province (26°21′ N; 98°49′ E) during its flourishing florescence on 9 November, 2016, and were inserted into deionized water before being transported to the Research Institute of Resources Insects, Chinese Academy of Forestry (RIRICAF) in Kunming. Subsequently, the inflorescences were preserved at 25 ± 1 °C. The flowers were classified into four groups according to their botanical characteristics (Figure 1) [4]: (I) bud stage: buds complete closed; (II) initial-flowering stage: semi-open petal; (III) full-flowering stage: completely open petals, observable yellow pistils and stamens; and (IV) end-flowering stage: petals and calyxes withered, stamens turned brown. Three replicates (three flowers from three inflorescences per replicate) were randomly conducted from different plants, and the results are means of three tests, including nine flowers.

### 3.2. Method

Volatile compounds of a complete flower were monitored using solid-phase microextraction (SPME). A total of five *L. yunnanensis* individuals were randomly selected and sampled for scent collection between 8:00 and 11:00; the method of scent collection was described by Li et al. [7]. The methods of scent collection and HS-SPME-GC-MS were consistent with the methods of *L. pinceana* [4] and *L. gratissima* [5]. Sampling by HS-SPME was performed by 100 µm polydimethylsiloxane (PDMS) SPME fiber. After the equilibration time (15 min), the fiber was exposed to the headspace of the capped glass vial (20 mL) to absorb volatile compounds for 40 min. The empty capped vial was used as the blank control.

A gas chromatograph-mass spectrometer (TRACE GC Ultra/ITQ900, Thermo Fisher Scientific, Inc., Waltham, MA, USA) coupled with a DB-5MS capillary column (5% diphenyl cross-linked 95% dimethylpolysiloxane, 30 m × 0.25 mm i.d. × 0.25 µm film thickness. Agilent J & W Scientific, Folsom, CA, USA) were used for the GC-MS analysis. The oven temperature was programmed at 40 °C for 2 min, increasing at a rate of 6 °C/min to 130 °C and then increasing at 15 °C/min to 280 °C for 5 min. The injector was performed in splitless mode for 1 min. Helium was the carrier gas at a flow rate of 1.0 mL/min. The mass spectrometer was operated in electron impact mode at an ionization voltage of 70 eV and ion source temperature of 250 °C, and the scan range was 50–650 amu.

### 3.3. Data Analysis

The GC-MS data were processed using the TF Xcalibur 2.1.0 software. Component identification was carried out using NIST 2008 mass spectral database and confirmed by the comparison of their linear retention index (*LRI*) with published data [15]. Identification of individual components could be confirmed by the comparison of both mass spectrum and GC retention data with those of authentic standards [32]. A series of n-alkane standards (C6-C19) (Accu Standard, New Haven, CT, USA) were analyzed under the same conditions to obtain *LRI* values for the volatile compounds. Peak areas were normalized as percentage and used to determine the relative amounts of the volatiles.

### 3.4. Statistical Analysis

The data were analyzed by one-way analysis of variance (ANOVA). Principal component analysis (PCA) and Bray–Curtis similarity (*BCS*) were carried out using PC-ORD for Windows (Version 5.0; MjM Software, Gleneden Beach, OR, USA).

## 4. Conclusions

HS-SPME coupled with GC-MS was adapted to determine the composition and content of floral scent emitted from different flower development stages of *L. yunnanensis*. The present investigation showed that 40 VOCs were identified in whole life flower-stages, and sesquiterpenes were the most abundant floral scent compounds. The amount of floral scent emission had the tendency to first ascend and descend in succession, reaching its peak level at the initial-flowering stage. However, the highest diversity of floral volatiles was detected at the full-flowering stage. Compared with the other two species of *Luculia* (*L. pinceana* and *L. gratissima*), the composition and relative content of floral scent were also different at four stages of the tree species. Furthermore, *L. yunnanensis* has a promising prospect for development as an essential oil source and for future breeding programs and cultivation, owing to the main compounds of the floral scent.

## Figures and Tables

**Table 1:** Volatile compounds identified in four different stages of *L. yunnanensis* flower development using headspace solid phase micro-extraction (HS-SPME) coupled with gas-chromatography mass-spectrometry (GC-MS) (HS-SPME-GC-MS). (I) bud stage; (II) initial-flowering stage; (III) full-flowering stage; and (IV) end-flower stage.

Peak	RT	*LRI*	*LRI* *	Compounds	CAS #	Relative Content (%) ± SD			
						I	II	III	IV
				**Monoterpenes**					
1	8.35	903	929	(1S)-2,6,6-Trimethylbicyclo[3.1.1]hept-2-ene	7785-26-4	0b	0b	0.25 ± 0.06a	00b
2	8.72	916	910	Santolina triene	2153-66-4	0b	0.01 ± 0.01b	0.16 ± 0.08a	00b
3	9.45	943	941	-α-Pinene	80-56-8	0b	0b	0.99 ± 0.20a	00b
4	9.81	957	966	β-Pinene	127-91-3	0b	0.09 ± 0.01b	0.07 ± 0.04b	0.42 ± 0.10a
7	11.32	1011	1011	3-Carene	13466-78-9	3.36 ± 0.44c	10.72 ± 2.20b	9.17 ± 1.65bc	29.18 ± 4.53a
8	12.63	1059	1056	*trans*-β-Ocimene	3779-61-1	0.88 ± 0.10b	0.61 ± 0.13b	0.99 ± 0.49b	1.86 ± 0.04a
11	13.81	1102	1127	α-Campholenal	91819-58-8	0b	0b	0.60 ± 0.30a	00b
12	14.37	1124	-	α-Santoline alcohol	90823-36-2	0b	0b	0.35 ± 0.18a	0b
13	14.84	1142	1137	Limonene oxide	1195-92-2	0.20 ± 0.14a	0a	0.28 ± 0.19a	0a
				**Aliphatics**					
5	10.64	987	987	Methyl heptenone	110-93-0	0.68 ± 0.26a	0b	0b	0b
6	11.14	1005	-	Furan, 2,3-dihydro-4-(1-methylethyl)-	34314-84-6	3.27 ± 0.89a	0.33 ± 0.02b	0.17 ± 0.03b	1.07 ± 0.05b
9	13.46	1089	1130	2,6-Dimethyl-1,3,5,7-octatetraene	460-01-5	0b	0b	0.29 ± 0.08a	0b
15	19.67	1340	1373	Propanoic acid, 2-methyl-, 2-ethyl-3-hydroxyhexyl ester	74367-31-0	1.12 ± 0.13a	0.25 ± 0.05b	0.22 ± 0.11b	1.16 ± 0.14a
40	28.08	1828	1827	Isopropyl myristate	110-27-0	0b	0b	0.18 ± 0.06b	1.51 ± 0.15a
				**Benzenoids**					
10	13.71	1098	1107	Phenylethyl alcohol	60-12-8	0b	0.10 ± 0.02a	0b	0b
14	18.31	1295	-	6,7-Dimethyl-1,2,3,5,8,8a -hexahydronaphthalene	107914-92-1	0b	0.14 ± 0.03a	0.17 ± 0.09a	0b
22	22.45	1433	1451	Paeonol	552-41-0	0b	0b	12.73 ± 2.43a	0b
36	24.99	1550	1550	Benzene, 1,2,3-trimethoxy-5-(2-propenyl)-	487-11-6	0c	0.41 ± 0.03a	0c	0.13 ± 0.07b
				**Sesquiterpenes**					
16	19.87	1346	1346	α-Cubebene	17699-14-8	13.39 ± 0.63a	10.24 ± 1.68b	8.68 ± 1.09b	7.52 ± 1.02b
17	20.34	1361	1365	α-Copaene	3856-25-5	16.59 ± 3.43a	17.92 ± 2.73a	9.30 ± 0.73b	7.45 ± 0.49b
18	21.13	1387	1377	Isoledene	95910-36-4	9.79 ± 1.21a	8.67 ± 0.65a	8.33 ± 1.94a	7.19 ± 1.40a
19	21.52	1399	1400	Caryophyllene	87-44-5	4.23 ± 0.49a	2.05 ± 0.12b	1.19 ± 0.26b	3.76 ± 0.69a
20	21.87	1412	1421	β-Ylangene	20479-06-5	0.60 ± 0.01a	0.34 ± 0.07b	0.08 ± 0.03c	0.36 ± 0.02b
21	22.22	1425	1438	α-Guaiene	3691-12-1	0b	0.12 ± 0.03a	0.04 ± 0.02b	0b
23	22.68	1441	1450	*cis*-Muurola-3,5-diene	Not available	3.43 ± 0.36ab	3.76 ± 0.50ab	4.48 ± 1.17a	2.61 ± 0.48b
24	22.81	1447	1446	Humulene	6753-98-6	3.89 ± 0.68b	1.70 ± 0.21c	3.42 ± 1.29bc	9.69 ± 0.49a
25	23.01	1454	1458	β-Gurjunene	17334-55-3	0b	0.23 ± 0.07a	0.04 ± 0.02a	0b
26	23.32	1466	1474	β-Cadinene	523-47-7	2.27 ± 0.06a	1.63 ± 0.20a	2.42 ± 0.64a	1.61 ± 0.55a
27	23.42	1469	1475	Naphthalene,1,2,4a,5,6,8a -hexahydro-4,7-dimethyl-1-(1-methylethyl)-	483-75-0	0b	0.66 ± 0.08a	0.08 ± 0.05b	0.77 ± 0.01a
28	23.56	1474	1474	γ-Muurolene	30021-74-0	3.15 ± 0.33b	4.50 ± 0.77a	1.79 ± 0.48c	1.95 ± 0.22bc
29	23.84	1485	1515	Cubebol	23445-02-5	4.36 ± 0.42a	3.89 ± 0.64a	3.85 ± 0.41a	2.52 ± 0.41b
30	23.97	1490	1484	-Aristolene	6831-16-9	1.05 ± 0.14a	0.83 ± 0.06ab	1.01 ± 0.41a	0.42 ± 0.02b
31	24.07	1493	1500	(E,E)-α-Farnesene	502-61-4	0.42 ± 0.02b	1.36 ± 0.26b	4.65 ± 1.13a	1.21 ± 0.07b
32	24.29	1503	1494	4-*epi*-Cubebol	Not available	4.24 ± 0.51b	9.06 ± 1.41a	4.67 ± 1.48b	2.90 ± 0.73b
33	24.44	1514	1519	δ-Cadinene	483-76-1	14.66 ± 2.85a	12.68 ± 2.56a	13.07 ± 2.79a	9.16 ± 0.77a
34	24.62	1525	1528	Cadine-1,4-diene	16728-99-7	5.74 ± 0.05ab	6.13 ± 0.73a	4.47 ± 1.86ab	3.13 ± 0.25b
35	24.87	1542	1542	α-Calacorene	21391-99-1	0c	0.32 ± 0.06a	0.18 ± 0.04b	0c
37	25.68	1596	1596	Guaiol	489-86-1	1.09 ± 0.14a	0.24 ± 0.07b	0.30 ± 0.04b	1.11 ± 0.27a
38	25.82	1608	1630	α-Acorenol	28296-85-7	0.72 ± 0.09a	0.30 ± 0.04b	0.33 ± 0.05b	0.73 ± 0.15a
39	26.32	1652	1647	Cubenol	21284-22-0	0.81 ± 0.01a	0.71 ± 0.02a	1.00 ± 0.43a	0.58 ± 0.01a
				**Monoterpenes**		**4.50 ± 0.41c (9.11%)**	**11.43 ± 2.31bc (20.21%)**	**12.86 ± 2.93b (22.78%)**	**31.46 ± 4.46a (14.18%)**
				**Aliphatics**		**5.07 ± 0.75a (14.79%)**	**0.58 ± 0.08c (13.79%)**	**0.86 ± 0.25c (29.07%)**	**3.74 ± 0.33b (8.82%)**
				**Benzenoids**		**0b**	**0.65 ± 0.07b (10.77%)**	**12.90 ± 2.35a (18.22%)**	**0.13 ± 0.07b (53.85%)**
				**Sesquiterpenes**		**90.43 ± 1.17a (1.29%)**	**87.34 ± 2.46a (2.82%)**	**73.38 ± 5.20b (7.09%)**	**64.67 ± 4.09b (6.32%)**

Values, expressed as mean ± SD of triplicate measurements, with different letter (a–c) in the same row indicating significant difference according to Tukey’s test (*p* < 0.05). The data in brackets are coefficients of variation. RT: retention time; *LRI*: linear retention index; *LRI* *: linear rentention index taken from NIST Standard Reference Database 69 [15]; CAS #: chemical abstracts service registry number.

**Table 2:** The Bray–Curtis similarity values (%) among stages of flower development of *L. yunnanensis*. (I) bud stage; (II) initial-flowering stage; (III) full-flowering stage; and (IV) end-flower stage.

	I	II	III	IV
I	100			
II	81.40	100		
III	71.88	76.30	100	
IV	63.71	65.67	64.29	100
